# CREB Inhibits AP-2α Expression to Regulate the Malignant Phenotype of Melanoma

**DOI:** 10.1371/journal.pone.0012452

**Published:** 2010-08-27

**Authors:** Vladislava O. Melnikova, Andrey S. Dobroff, Maya Zigler, Gabriel J. Villares, Russell R. Braeuer, Hua Wang, Li Huang, Menashe Bar-Eli

**Affiliations:** Department of Cancer Biology, The University of Texas M. D. Anderson Cancer Center, Houston, Texas, United States of America; Universidade de São Paulo, Brazil

## Abstract

**Background:**

The loss of AP-2α and increased activity of cAMP-responsive element binding (CREB) protein are two hallmarks of malignant progression of cutaneous melanoma. However, the molecular mechanism responsible for the loss of AP-2α during melanoma progression remains unknown.

**Methodology/Principal Findings:**

Herein, we demonstrate that both inhibition of PKA-dependent CREB phosphorylation, as well as silencing of CREB expression by shRNA, restored AP-2α protein expression in two metastatic melanoma cell lines. Moreover, rescue of CREB expression in CREB-silenced cell lines downregulates expression of AP-2α. Loss of AP-2α expression in metastatic melanoma occurs via a dual mechanism involving binding of CREB to the AP-2α promoter and CREB-induced overexpression of another oncogenic transcription factor, E2F-1. Upregulation of AP-2α expression following CREB silencing increases endogenous p21*^Waf1^* and decreases MCAM/MUC18, both known to be downstream target genes of AP-2α involved in melanoma progression.

**Conclusions/Significance:**

Since AP-2α regulates several genes associated with the metastatic potential of melanoma including c-KIT, VEGF, PAR-1, MCAM/MUC18, and p21*^Waf1^*, our data identified CREB as a major regulator of the malignant melanoma phenotype.

## Introduction

Malignant melanoma is a highly aggressive disease for which successful treatment modalities have not been established. A number of genetic alterations including loss of tumor suppressor proteins encoded by the INK4a/ARF gene locus, or oncogenic BRAF mutations, occur early during melanomagenesis [1], [2], [3]. However, the key event in the progression of melanoma toward a malignant, locally-invasive and metastatic phenotype is the loss of the Activator Protein-2α (AP-2α) tumor suppressor protein.

AP-2α is a 52-kD retinoic acid-inducible protein that regulates gene expression during embryonic morphogenesis and adult cell differentiation [4], [5]. In cancers like melanoma, AP-2α and other de-regulated transcription factors, including Activating Transcription Factor-1/2 (ATF-1/2)/cAMP-responsive Element Binding (CREB) protein, SNAIL/SLUG, nuclear factor kappa B (NFκB), signal transducers and activators of transcription (STAT) 3 and 5, directly control the expression of adhesion molecules, matrix-degrading enzymes, motility factors, cytokines, survival factors and growth factors/receptors enabling complex interactions between melanoma cells and the extracellular milieu during metastatic dissemination [6], [7], [8], [9], [10], [11], [12], [13].

Animal model studies helped demonstrate that inactivation of AP-2α in SB2 non-metastatic primary cutaneous melanoma cells by dominant-negative AP-2B, augmented melanoma cell tumorigenicity [14]. Inversely, an enforced overexpression of AP-2α in metastatic melanoma cells inhibited tumor cell growth at subcutaneous sites and abrogated development of melanoma lung metastasis *in vivo* [15]. Importantly, we have shown through melanoma tissue microarrays and quantitative immunofluorescence analysis, that AP-2α expression correlates with poor prognosis in melanoma patients [11], [16]. In many tumor types, including melanoma, AP-2α acts as a tumor suppressor by activating p21*^Waf1/Cip1^* expression and inducing cell cycle arrest [17], [18], [19]. Loss of AP-2α in metastatic melanoma is directly linked to overexpression of melanoma cell adhesion molecule MCAM/MUC18, protease-activated receptor-1 (PAR-1), matrix metalloproteinase-2 (MMP-2), and loss of tyrosine-kinase receptor c-KIT [10], [11], [12], [14], [15], [20].

Restoration of AP-2α in colon cancer cells reduced tumor burden in mice and inhibited spontaneous liver metastasis by increasing the E-cadherin/MMP-9 ratio [21]. Overexpression of AP-2α in pancreatic cancer cells was shown to reduce tumor growth through an altered expression pattern of cell cycle-controlling factors such as CDK-4, CDK-6, Cyclin-G1, p27^kip1^ and p57^kip2^ [22]. In various cell models, AP-2α has also been shown to regulate c-erbB-2/HER2-2/neu, plasminogen activator inhibitor type I (PAI-1), insulin-like growth factor binding protein-5 (IGFBP-5), transforming growth factor-α (TGF-α), hepatocyte growth factor (HGF), vascular endothelial growth factor (VEGF), and c-Myc [23], [24], [25], [26], [27], [28], [29], [30], [31]. Despite an improved understanding of its mechanisms of action, the cause for the loss of AP-2α in melanoma remains unknown.

Previous studies have suggested an inverse correlation between loss of the tumor suppressor AP-2α and upregulation of CREB expression and activity with melanoma progression [32], [33]. CREB is a member of a leucine zipper class of transcription factors that binds to cAMP-response elements (CREs) found within the promoter and enhancer regions of hundreds of genes [34]. CREB is activated by a number of growth factors, hormones and stress signals that trigger its phosphorylation at Ser133 and its association with the co-activator paralogs CBP and p300 [35], [36]. Co-activators such as TORC2, ACT and TAF4 may stabilize the assembly of co-activator complexes over CREB target genes in specific cell types thereby regulating CREB activity [34], [35], [36], [37].

CREB regulates the expression of genes that suppress apoptosis, induce cell proliferation and mediate inflammation and tumor metastasis such as BCL-2, HER-2, IL-8 and MMP-2 [8], [38], [39]. In melanoma, inactivation of CREB by dominant-negative KCREB reduced tumor growth and experimental lung metastasis by acting as a survival factor and regulating the expression and activity of MMP-2 and the adhesion molecule MCAM/MUC18 [37], [40], [41], [42]. We have demonstrated that activation of CREB phosphorylation in melanoma is associated with constitutive activation of the pro-inflammatory G-protein coupled receptors PAR-1 and Platelet-Activating Factor Receptor [43]. Most recently, we found that CREB acts as a negative regulator of the melanoma tumor suppressor gene cysteine-rich protein 61 (*CCN1/CYR61*) [7].

An overlap in the spectrum of target genes regulated by AP-2α and CREB led us to explore the connection between the two transcription factors. Herein, we propose a potential mechanism by which CREB induces transcriptional repression of AP-2α during melanoma progression through upregulation of oncogenic E2F-1. Since AP-2α regulates key genes associated with the acquisition of the metastatic phenotype, our observation that CREB regulates the AP-2α expression emphasizes its role as a “master switch” in melanoma progression.

## Results

### Loss of AP-2α expression correlates with metastatic potential of melanoma cell lines

**Figure 1:**
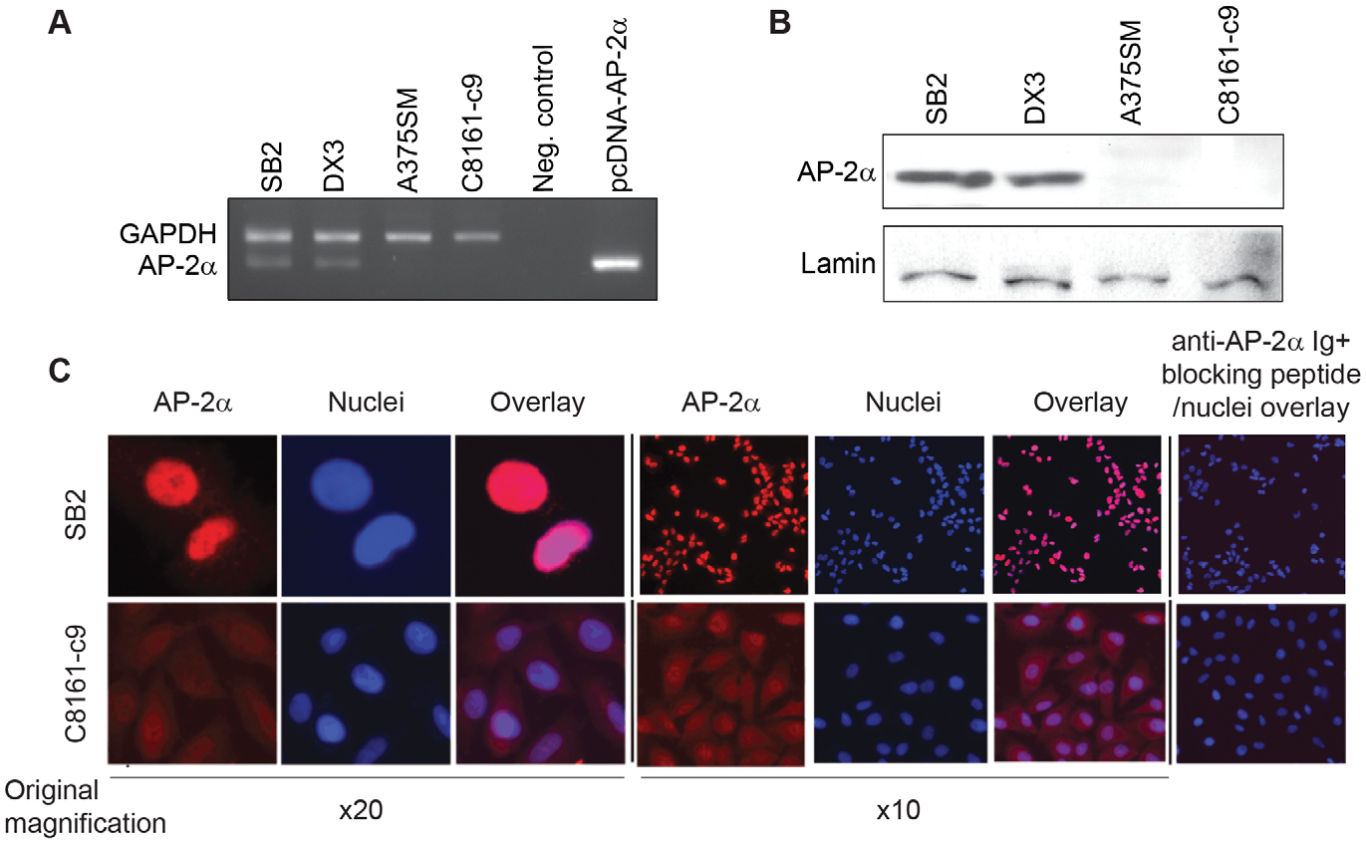
Expression of AP-2**α** inversely correlates with melanoma metastatic potential. (A) Expression of AP-2α mRNA in human melanoma cell lines was detected by reverse transcriptase-PCR (RT-PCR). High levels of AP-2α expression are observed in SB2 and DX3 (non-metastatic cell lines) whereas A375SM and C8161-c9 (metastatic cell lines) show undetectable levels of AP-2α. The pcDNA3.1 AP-2α was used as a positive control. Glyceraldehyde-3-phosphate dehydrogenase (GAPDH) mRNA was used as a housekeeping gene. (B) Western Blot analysis of nuclear extracts isolated from subconfluent cultures and analyzed for AP-2α protein expression. Similarly, AP-2α protein is detected in non-metastatic cell lines. In metastatic cell lines, AP-2α expression was found to be downregulated by 99%. Lamin was used a loading control. (C) SB2 and C8161-c9 cell lines were immunofluorescently stained for detection of AP-2α (Red). Colocalization with DAPI (Blue) and AP-2α reveal high AP-2α intensity in the nuclei of SB2 cells while low levels of AP-2α are detected dispersed within the cytoplasm of the C8161-c9 cell.

To study the mechanism of AP-2α loss during melanoma progression, we first examined the status of AP-2α by RT-PCR in a panel of human melanoma cell lines exhibiting different metastatic capabilities. Figure 1A demonstrates that AP-2α is expressed in the non-metastatic cell lines SB2 and DX3 while both A375SM and C8161-c9 metastatic melanoma cell lines do not express detectable levels of AP-2α mRNA. Furthermore, AP-2α protein expression levels were determined by Western Blot. Similarly, AP-2α was detectable in the nucleus of SB2 and DX3 whereas AP-2α was absent in both A375SM and C8161-c9 (Figure 1B).

We have previously shown that loss of nuclear AP-2α and an increase in the cytoplasmic to nuclear AP-2α ratio correlates with poor prognosis of melanoma patients [11], [16]. To confirm if this phenotype is recapitulated in melanoma cell lines, we examined subcellular localization of AP-2α in cultured cell lines by immunofluorescense. Consistent with our previous finding in human melanoma specimens, we found that AP-2α protein is expressed in the nuclei of non-metastatic SB2 cells but is nearly absent in metastatic C8161-c9 cells (Figure 1C). This observation was confirmed in a panel of 9 additional cell lines with varying metastatic potential (data not shown).

### CREB phosphorylation correlates with the metastatic potential of melanoma cell lines and inversely correlates with AP-2α expression

**Figure 2:**
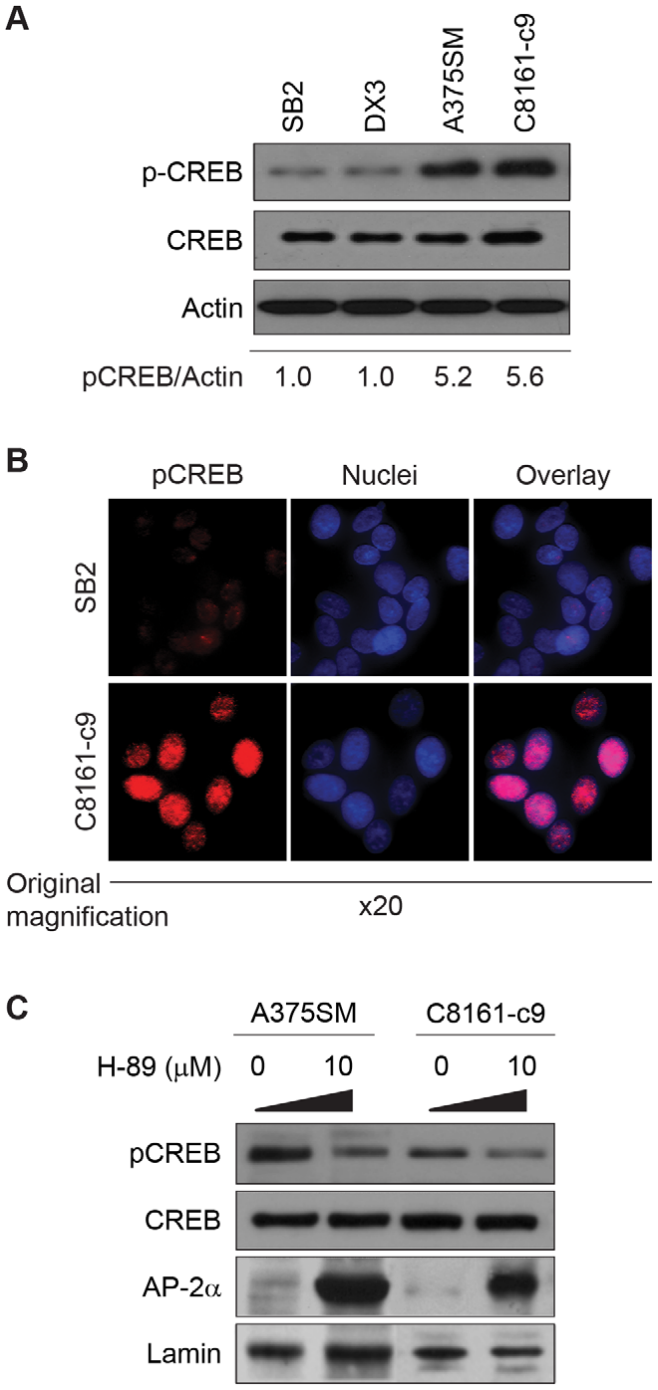
Inhibition of pCREB results in increased AP-2α expression. (A) pCREB was analyzed in non-metastatic and metastatic cell lines by Western Blot. As confirmed by densitometry, pCREB in metastatic cell lines is approximately 5-fold higher as compared to non-metastatic cell lines. α-Actin is used as a loading control. (B) SB2 and C8161-c9 cell lines were immunofluorescently stained for detection of pCREB (Red). Colocalization with DAPI (Blue) and pCREB reveal high pCREB intensity in the nuclei of C8161-c9 cells (low AP-2α) while low level of pCREB is mostly undetectable in the nuclei of SB2 cells (high AP-2α). (C) Expression of AP-2α was detected in A375SM and C8161-c9 cell lines after incubation with the pCREB inhibitor, H-89. Complete restoration of AP-2α expression was observed in both cell lines after treatment with H-89 at 10 mM. Lamin was used as a loading control.

Several factors involved in melanoma invasion, resistance to apoptosis and proliferation, such as MMP-2, MCAM/MUC18, BCL-2 and TGF-α are regulated by both CREB and AP-2α, albeit in an opposite manner [8], [33], [37]. Therefore, we tested the hypothesis that a gain in CREB activity results in the loss of AP-2α expression in melanoma. Analysis of CREB protein expression by Western Blot demonstrates that while levels of total CREB protein remain similar in all melanoma cell lines, the levels of its phosphorylation at Serine 133 (pCREB), a critical functional phosphorylation site, were significantly higher (approximately 5-fold increase) in metastatic melanoma cell lines (A375SM and C8161-c9) as compared to non-metastatic melanoma cell lines (SB2 and DX3) (Figure 2A). Additionally, we assessed the levels of pCREB by confocal microscopy in both SB2 and C8161-c9. As depicted in Figure 2B, high pCREB intensity in the nuclei of C8161-c9 cells (low AP-2α) was detected whereas pCREB was mostly undetectable in the nuclei of SB2 cells (high AP-2α), thus supporting our Western Blot analyses.

To examine whether activation of CREB results in loss of AP-2α expression, we inhibited CREB phosphorylation in A375SM and C8161-c9 cells by using several kinase inhibitors such as H-89 (PKA/MSK pathway), KN-93 (CaMKIV pathway), SL0101-1 (p90RSK pathway), and Triciribine (Akt pathway). H-89 restored the overall levels of AP-2α expression (Figure 2C), and induced accumulation of AP-2α in the nuclei of A375SM and C8161-c9 cells (data not shown). KN-93, SL0101-1 and Triciribine inhibited CREB phosphorylation, in a similar manner to H-89, leading to increased expression of AP-2α (Figure S1), suggesting that activation of CREB during metastatic progression results in loss of AP-2α expression.

### CREB acts as a negative transcriptional regulator of AP-2α expression

**Figure 3:**
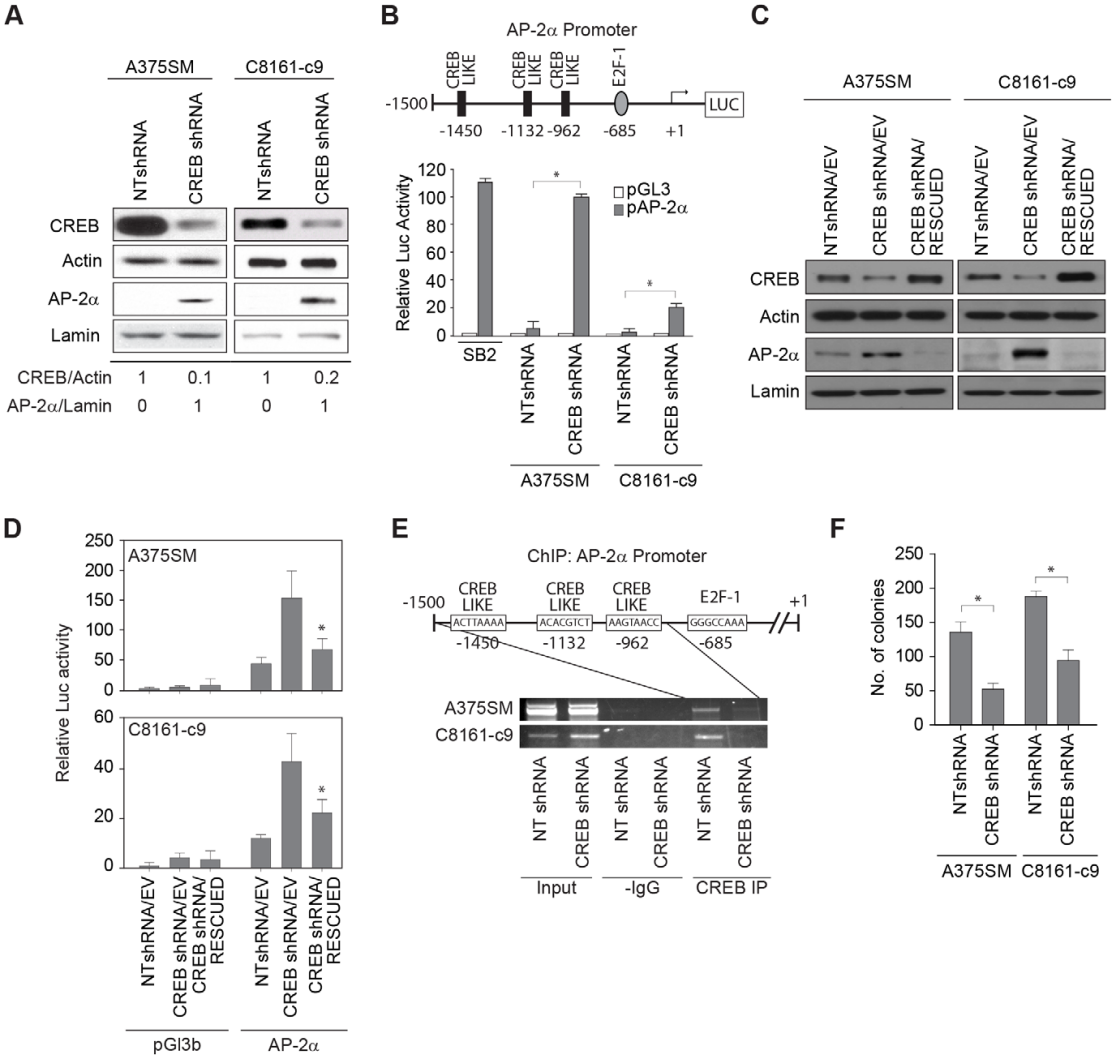
Silencing CREB increases AP-2α expression and promoter activity. (A) Western Blot analysis showing silencing of CREB in both A375SM and C8161-c9 cells stably transduced with CREB shRNA as compared to non-targeting control (NTshRNA). Approximately 80–90% CREB downregulation was observed in the CREB shRNA transduced cells as compared to NTshRNA as determined by densitometry. Following silencing of CREB, expression levels of AP-2α were upregulated in both A375SM and C8161-c9 cell lines as detected by Western Blot. α-Actin was used as a loading control for total lysate samples. Lamin is used as a loading control for nuclear fractions. (B) The AP-2α promoter region (nucleotides −1,500 to +50 from the transcription initiation site) was amplified from genomic DNA and cloned into the pGL3-basic firefly luciferase vector. Schematic representation of the promoter region shows the predicted CREB and E2F-1 binding sites. The luciferase activity driven by the AP-2α promoter was significantly increased by 90- and 20-fold after CREB silencing in both A375SM and C8161-c9 cell lines respectively, as compared to NT transduced cells. *, *p*<0.001. SB2 cells were used as a positive control. (C) Rescue of CREB expression in CREB-silenced cells resulted in down-regulation of AP-2α expression. α-Actin was used as a loading control. (D) The luciferase activity driven by the AP-2α promoter decreased significantly (*, *p*<0.001) after rescue of CREB expression in both A375SM and C8161-c9 cell lines. NTshRNA/EV, nontargeting control cells. CREBshRNA/EV, CREB-silenced control cells. CREBshRNA/RESCUED, CREB-silenced cells transduced with CREB nontargetable expression vector. (E) Chromatin Immunoprecipitation (ChIP) studies showed decreased binding of CREB to the promoter of AP-2α in both CREB-silenced cell lines (A375SM and C8161-c9) as compared to NT transduced cell lines. IgG antibodies were used as negative controls. Input DNA was used to determine equal amounts of chromatin. (F) Colony formation assay performed in soft agar layer depicts a significant decrease in clonogenicity after silencing CREB in both A375SM and C8161-c9. *, *p*<0.001.

To confirm that restoration of AP-2α is specific to CREB, we stably silenced CREB expression in metastatic A375SM and C8161-c9 cells lines using lentiviral small hairpin RNA (shRNA), as described previously [7]. Figure 3A demonstrates that CREB expression was reduced by 90% and 80% in A375SM and C8161-c9 cells respectively, as compared to cells transduced with non-targeting shRNA (NTshRNA). In line with the use of pathway inhibitors to decrease pCREB, CREB silencing via shRNA resulted in upregulation of AP-2α expression in both metastatic melanoma cell lines, confirming that CREB indeed acts as a negative regulator of AP-2α (Figure 3A).

We next analyzed the promoter region (2Kb) of the human AP-2α gene (GenBank Accession Number NM_003220.2). AP-2α promoter analysis for consensus transcription factor binding motifs using Genomatix software revealed the presence of three previously unidentified, putative CRE-like sites at positions −1,450 bp (ACTTAAAA), −1,132 bp (ACACGTCT) and −962 bp (AAGTAACC) of the proximal AP-2α promoter region (Figure 3B - Schematic representation). In addition, we also found one binding site for oncogenic transcription factor E2F-1 at position -685bp (GGGCCAAA) of the proximal AP-2α promoter (Figure 3B). To confirm that CREB regulates AP-2α expression at the transcriptional level, we cloned the AP-2α promoter in front of a luciferase reporter gene. Reflecting the pattern of AP-2α mRNA expression (Figure 1A), AP-2α promoter activity was significantly higher in non-metastatic SB2 cells as compared to metastatic A375SM and C8161-c9 cells transduced with NTshRNA (Figure 3B). The luciferase activity driven by the AP-2α promoter increased after CREB silencing by 90-fold (*p*<0.001) in A375SM and by 20-fold (*p*<0.001) in C8161-c9 cells as compared to the activity in NTshRNA control cells (Figure 3B).

To further establish the link between CREB and AP-2α expression, we next rescued CREB expression in both CREB-silenced cell lines by overexpressing CREB in which silent mutations were introduced to render it nontargetable to CREB shRNA (Figure 3C). Western Blot analysis of these cells demonstrated that CREB rescue resulted in restoration of CREB expression to levels comparable to those in control cells (NTshRNA/EV). Restoring CREB expression in these cells also resulted in decreased AP-2α expression (Figure 3C) and decreased luciferase activity driven by the AP-2α promoter (Figure 3D). Moreover, we sought to confirm that inhibition of AP-2α was dependent on CREB phosphorylation. To that end, we created a missense mutation at position 133 (S → A), the main phosphorylation site of CREB [34], in the nontargetable expression vector (CREB_(S133A)_). Western Blot analysis of these cells revealed that CREB_(S133A)_ expression vector rescued the total level of CREB. However, this mutant was not able to decrease the AP-2α expression in either of the CREB-silenced cell lines (Figure S2).

To confirm that CREB binds to the AP-2α promoter, we next utilized Chromatin Immunoprecipitation (ChIP) with primers designed to amplify the AP-2α gene 5′ flanking sequence that contains 3 putative CRE-like binding sites (Figure 3E). We found that CREB directly associates with AP-2α promoter in A375SM and C8161-c9 NTshRNA cells. This binding was significantly decreased after CREB silencing in both cell lines (Figure 3E).

Additionally, both CREB and AP-2α have been shown to regulate expression of genes involved in cell proliferation [8]. We have previously demonstrated that overexpression of AP-2α in colon cancer cells reduces their ability to form colonies in agar [21]. Therefore, in order to understand the contribution of CREB to melanoma growth and metastasis, we next assessed the effect of CREB silencing on the clonogenicity of metastatic melanoma cells. As seen in Figure 3F, both A375SM and C8161-c9 stably transduced with CREB shRNA had a significant decrease in size and number of colonies after 25 days as compared to NTshRNA cells. The mean number of colonies decreased significantly by 62% in A375SM cells (*p*<0.001), and by 49% in C8161-c9 cells, (*p*<0.001). Taken together, our data suggest that CREB regulates AP-2α transcription in melanoma cells via direct binding to its promoter, thus repressing AP-2α expression and its tumor suppressor role.

### E2F-1 acts as a negative regulator of AP-2α promoter activity and expression downstream of CREB

**Figure 4:**
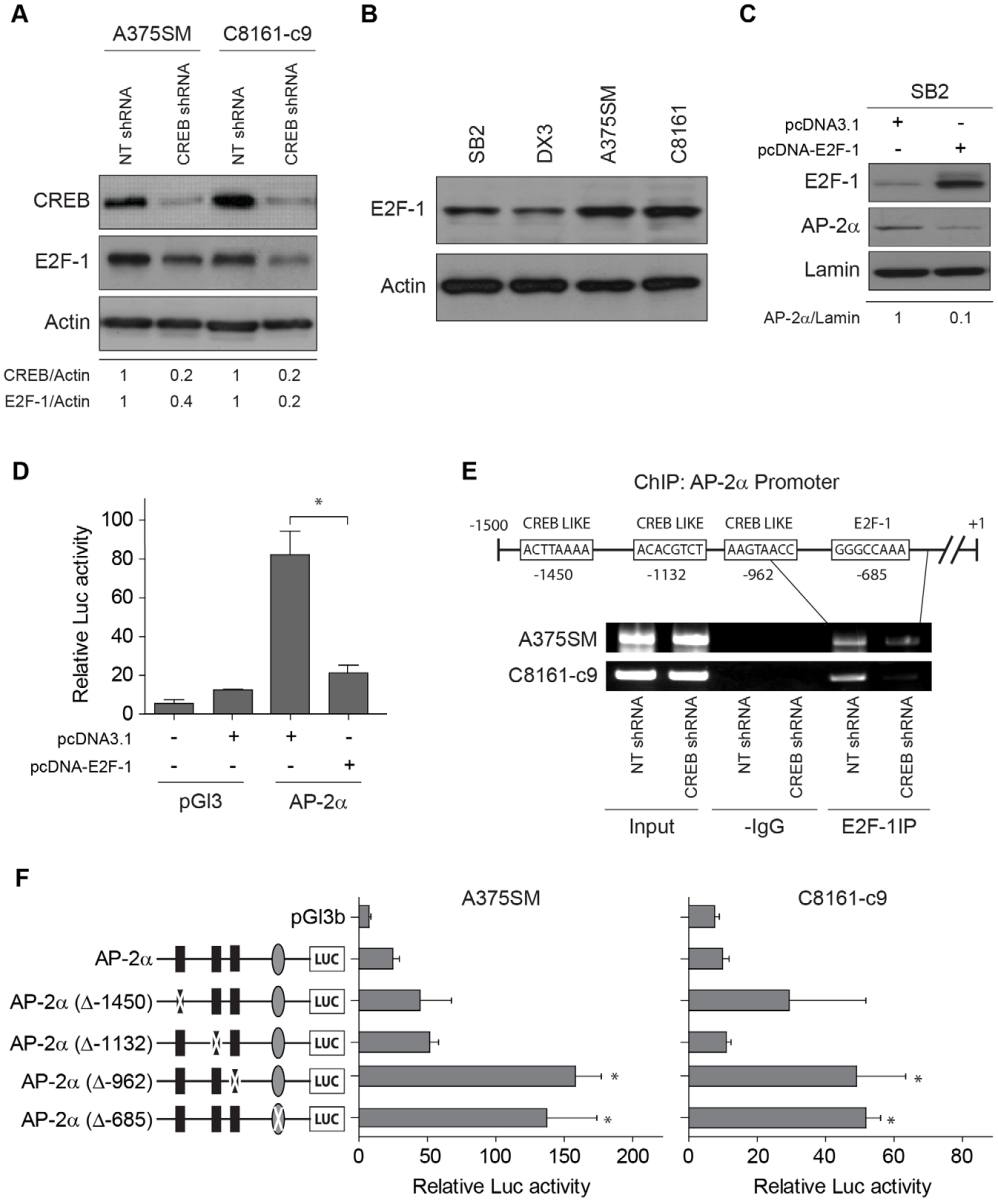
E2F-1 regulates AP-2α transcription by direct binding to the AP-2α promoter. (A) Western Blot analysis showing downregulation of E2F-1 in both CREB-silenced A375SM and C8161-c9 cells by 60% and 80% respectively, as compared to non-targeting control (NTshRNA). Actin is used as a loading control. (B) E2F-1 expression was analyzed by Western Blot in SB2, DX3 (low metastatic), A375SM and C8161-c9 (highly metastatic) total cell extracts. Lower expression of E2F-1 is detected in non-metastatic cell lines while in metastatic cell lines, E2F-1 is upregulated. Actin was used a loading control. (C) Transient expression of E2F-1 significantly decreased AP-2α protein levels in SB2 cells by 90%. (D) The luciferase activity driven by the AP-2α promoter was significantly decreased after transient expression of E2F-1 in the SB2 cell line (high AP-2α, low E2F-1 expression) as compared with pcDNA3.1 alone. *, *p*<0.001. (E) Chromatin Immunoprecipitation (ChIP) assays showed decreased binding of E2F-1 to the AP-2α promoter in both CREB-silenced cell lines (A375SM and C8161-c9) as compared to NT transduced cell lines. IgG antibodies were used as negative controls. Input DNA was used to determine equal amounts of chromatin. (F) A schematic representation of the promoter point mutations is depicted on the left side of the panel. The three identified CRE-like binding sites as well as the E2F-1 biding site were mutated, as described in “Material and Methods”. Mutation of one CREB-like binding site (−962) as well as the E2F-1 binding site, led to a significant increase in the AP-2α promoter activity in both the A375SM and C8161-c9 parental cells. *p,<0.01.

We have previously subjected CREB-silenced A375SM cells to cDNA microarray analyses in order to identify downstream target genes regulated by CREB in melanoma [7]. cDNA microarray experiments have indicated that E2F-1 is one of the genes whose expression was decreased by CREB silencing. Since multiple E2F-1 binding sites are present on the promoter of AP-2α, we investigated the hypothesis that E2F-1 acts downstream of CREB and that together, CREB and E2F-1 regulate AP-2α expression. Indeed, CREB silencing led to decreased E2F-1 protein expression in both cell lines by 60% (A375SM) and 80% (C8161-c9), thus confirming our cDNA microarray findings (Figure 4A).

To assess the status of E2F-1 during melanoma progression, we performed Western Blot analysis in non-metastatic and metastatic melanoma cell lines. As shown in Figure 4B, E2F-1 was found to be upregulated in A375SM and C8161-c9 as compared to non-metastatic cell lines, SB2 and DX3. To investigate whether E2F-1 contributes to regulation of AP-2α expression, we transiently transfected E2F-1 into an AP-2α-positive SB2 cell line. E2F-1 overexpression resulted in significant downregulation of AP-2α protein expression by 90% in SB2 cells (Figure 4C). As expected, overexpression of E2F-1 in AP-2α-negative A375SM cells did not affect AP-2α levels (data not shown). Loss of AP-2α protein in SB2 cells was further paralleled by a significant, 4-fold decrease in AP-2α promoter activity upon overexpression of E2F-1 (Figure 4D, *p*<0.001).

To confirm that E2F-1 regulates AP-2α transcription via direct binding to its promoter, we conducted another series of ChIP experiments using ChIP primers situated close to the E2F-1 binding site. We found that in metastatic melanoma cells, E2F-1 actively binds the AP-2α promoter. Furthermore, CREB silencing inhibited E2F-1 binding to the AP-2α promoter in both A375SM and C8161-c9 cells (Figure 4E).

Finally, to verify that the CREB-like sites as well as the E2F-1 site within the AP-2α promoter were involved in the regulation of AP-2α transcription, we altered each binding site by introducing point mutations. Mutation of a CREB element at position -962 and a E2F-1 element at position -685 (Figure 4F) led to a significant increase in the AP-2α promoter-driven reporter activity in both A375SM and C8161-c9 parental cells (Figure 4F). Interestingly, when analyzing CREB-like sites at position –1450 and –1132, no significant differences in the reporter activity were found (Figure 4F). Overall, these site-directed mutation analyses revealed that the CREB site located at -962 and the E2F-1 site located at −685 within the AP-2α promoter region, are essential for regulating AP-2α transcription.

Taken together, our results suggest that CREB regulates AP-2α expression via two pathways: 1) by directly binding the AP-2α promoter and repressing its activity, and 2) by stimulating expression of E2F-1, which also binds to AP-2α promoter and represses its activity.

### Increase in AP-2α restores the non-metastatic phenotype of melanoma by regulating p21*^Waf1^* and MCAM/MUC18

Since AP-2α regulates several genes involved in the progression of human melanoma [12], [15], [33], we sought to determine if re-expression of AP-2α after CREB silencing reverts the expression of AP-2α downstream target genes.

It has been previously shown that forced expression of AP-2α resulted in a transient transcriptional upregulation of p21*^Waf1^* expression and concomitant cell cycle arrest [19]. Furthermore, we have shown that upregulation of MCAM/MUC18 expression in metastatic cells correlates with loss of expression of AP-2α. Indeed, we have also shown that AP-2α regulates MCAM/MUC18 expression through direct binding to its promoter, suppressing tumorigenicity and metastatic potential of human melanoma cells [12].

**Figure 5:**
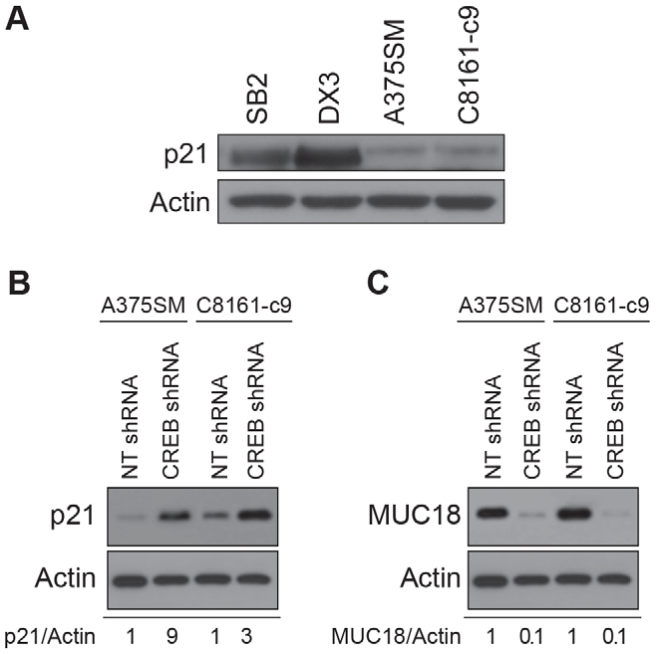
Upregulation of AP-2α in metastatic melanoma after CREB silencing modulates p21*^Waf1^* and MCAM/MUC18 expression. (A) Expression of p21*^Waf1^* in a panel of melanoma cell lines with different metastatic potential. (B) p21*^Waf1^* expression was upregulated in the two CREB-silenced A375SM and C8161-c9 cell lines. (C) MCAM/MUC18 was downregulated by 90% in both A375SM and C8161-c9 cell lines following CREB silencing. Actin was used as a loading control.

Figure 5A demonstrates that p21*^Waf1^* is expressed in the non-metastatic SB2 and DX3 cell lines but it is downregulated in the metastatic melanoma cell lines A375SM and C8161-c9. Additionaly, the CREB-silenced A375SM and C8161-c9 cell lines exhibited upregulation of p21*^Waf1^* expression by 9- and 3-fold respectively, as compared to cells transduced with NT shRNA (Figure 5B). On the other hand, MCAM/MUC18, which has been shown to be upregulated in metastatic melanoma cell lines [43], [44], [45], was found to be significantly downregulated by 90% in both cell lines after CREB silencing (Figure 5C).

Altogether, these experiments uncover the link between AP-2α and CREB, thereby establishing a new molecular mechanism by which CREB induces transcriptional repression of AP-2α via upregulation of E2F-1. These studies provide yet another mechanism by which CREB contributes to the acquisition of the malignant phenotype in human melanoma.

## Discussion

Melanoma progression is associated with several molecular changes including CCN1/CYR61, MCAM/MUC18 and PAR-1 [7], [8], [44], [46]. We have shown that one of the major switches related with melanoma progression is the loss of expression of the transcription factor AP-2α [15]. However, the molecular mechanism by which AP-2α is downregulated during melanogenesis has not been elucidated.

In the early 80s and mid 90s, several studies showed that the lack of expression or downregulation in AP-2α in malignant melanoma cells could be explained through a deletion of the distal portion of the long arm of chromosome 6 [47], [48], [49] or with abnormalities in the short arm of chromosome 6 (6p), near the HLA locus to which the AP-2α gene is mapped [48], [50]. Although, re-introduction of chromosome 6 into metastatic melanoma cells inhibits their tumorigenicity and metastatic potential [51], [52], [53], the status of AP-2α was not clarified. Thus, we previously showed by quantitative analysis of melanoma tissue microarrays, that the loss of nuclear AP-2α expression was associated with malignant transformation and progression of melanoma and that high cytoplasmic to nuclear ratio of AP-2α correlates with poor prognosis [11], [16]. Corroborating the tissue microarray data, our current study further reveals an inverse correlation between AP-2α expression and the metastatic potential of melanoma cell lines [11], [16], [54].

AP-2α plays a pivotal role in regulating the expression of several genes involved in cell proliferation (HER2), cycle regulation (p21*^Waf1^*), apoptosis (c-KIT, Bcl-2, FAS/APO-1), adhesion (E-cadherin), and invasion/angiogenesis (MMP-2, VEGF) [8], [15], [33]. Recently, overexpression of AP-2α in pancreatic cancer cells was shown to reduce tumor growth through an altered expression pattern of cell cycle-controlling factors such as CDK-4, CDK-6, cyclin-G1, p27^kip1^ and p57^kip2^ [22]. Additionally, some of the genes regulated by AP-2α in melanoma, such as MCAM/MUC18 and MMP-2, are also regulated by the CREB transcription factor [8], [37]. Interestingly, we demonstrated that AP-2α is downregulated with a concomitant increase in CREB expression during melanoma progression [55]. With that in mind, we have shown that inactivation of AP-2α by a dominant-negative AP-2α (AP-2B) in SB2 non-metastatic cells increased cell tumorigenicity in nude mice [14]. Moreover, overexpression of AP-2α in metastatic melanoma cells reduced tumor growth and experimental lung metastasis through downregulation of the above described factors [20].

In our current study, inhibition of p-CREB by H-89 significantly restored the levels of AP-2α as well as its translocation to the nucleus, thus leading us to hypothesize that CREB is involved in the regulation of AP-2α in melanoma. To study the mechanism by which CREB regulates AP-2α expression, we stably transduced metastatic melanoma cell lines with shRNA targeting CREB. Similar to treatment with H-89, silencing CREB led to significant upregulation of AP-2α expression. In addition, the activity of the luciferase reporter gene driven by the AP-2α promoter (−1,500 bp to +50 bp) was increased after CREB silencing. Previous work has shown that the promoter of AP-2α does not contain any canonical sequence motifs for basal transcription factors, such as TATA-, CCAAT or SP-1 boxes [56]. Moreover, initiation of transcription occurs just upstream of a long TC-rich region between residues −240 and −100. Further analyses also showed that the promoter of the AP-2α gene is subject to positive autoregulation by its own gene product [56]. Furthermore, our analyses of the AP-2α promoter revealed putative consensus binding sites for CREB and E2F-1 transcription factors.

Herein we demonstrate that the expression levels of E2F-1 were also downregulated after CREB silencing in both A375SM and C8161-c9 cell lines. However, it had not been described whether CREB regulates E2F-1 expression at the transcriptional level during melanoma progression. The E2F transcription-factor family is inextricably linked with cell-cycle control and apoptosis. E2F coordinates a large group of genes involved in regulating the G1-to S-phase transition, as well as other genes involved in apoptosis [57], [58], [59]. Conversely, studies on the well characterized E2F-1 have indicated that it may have characteristics of both an oncogene, by promoting the proliferation of cells beyond their normal constraints, and as a tumor suppressor [60], [61], [62], [63]. Recently, silencing E2F-1 effectively inhibited gastric cancer progression by reducing cell proliferation and increasing apoptosis [64] suggesting an oncogenic function.

Our ChIP analyses provide additional mechanistic evidence that CREB and E2F-1 negatively affect AP-2α transcription by binding to and inhibiting the activity of its promoter. In fact, forced expression of E2F-1 in SB2 cells decreased the expression of AP-2α as well as the luciferase activity driven by the AP-2α promoter. This data suggests that E2F-1 itself can suppress AP-2α transcription thereby uncovering a new potent target for melanoma therapy.

Finally, we confirmed that the upregulation of AP-2α after CREB silencing was functional and could modulate the expression of p21*^Waf1^* and MCAM/MUC18. AP-2α has been previously shown to activate p21*^Waf1^* leading to inhibition of cellular DNA synthesis and stable colony formation in colon carcinoma cells [17]. Moreover, significant correlation between AP-2α expression and p21*^Waf1^* levels have been reported in breast cancer and stage I cutaneous malignant melanoma [65], [66]. We have previously shown that the level of expression of MCAM/MUC18 in melanoma directly correlates with tumor progression and the acquisition of metastatic potential as it is transcriptionaly regulated by both AP-2α and CREB [12]. Our data show a significant upregulation in p21*^Waf1^* expression along with decreased MCAM/MUC18 expression following CREB silencing and AP-2α expression thereby confirming functional modulation of AP-2α by CREB.

Taken together, we provide a novel molecular mechanism by which oncongenic transcription factors, CREB and E2F-1, contribute to the metastatic phenotype of melanoma by negatively regulating AP-2α expression at the transcriptional level.

## Materials and Methods

### Cell lines, culture conditions and treatment

All the cell lines used in this study are established cell lines. The A375SM human melanoma cell line [67] was previously established from pooled lung metastases produced by A375-P cells injected i.v. into nude mice and maintained in Eagle's MEM supplemented with 10% fetal bovine serum (FBS), as previously described [20]. Both non-metastatic cell lines, SB2 [68] and DX3 [69], were maintained in Eagle's MEM supplemented with 10% FBS. The amelanotic C8161-c9 aggressive human melanoma cell line [70] was maintained in DMEM-F12 supplemented with 5% FBS. The 293FT cells (Invitrogen), used to produce lentiviral shRNA were maintained in DMEM supplemented with 10% FBS, according to the manufacturer's instructions. For experiments utilizing H-89 (Calbiochem), KN-93 (Sigma-Aldrich), SL0101-1 (TOCRIS Bioscience) or Triciribine (Sigma-Aldrich) inhibitors, the cells were deprived of serum for 24 h and then treated with IC_50_ of 10 µM, 0.5 µM, 1 µM and 10 µM respectively, for 24 h.

### Lentiviral shRNA to CREB

CREB shRNA (target sequence: 5′-GAGAGAGGTCCGTCTAATG-3′) and a non-targeting shRNA (target sequence: 5′-UUCUCCGAACGUGUCACGU-3′) were obtained as previously described [7]. Metastatic melanoma A375SM and C8161-c9 cell lines plated at 70% confluency in six-well plates were transduced with the virus. After 16 hours, the virus-containing medium was removed and replaced with normal growth medium [7].

### Nontargetable CREB Expression Vector

The lentiviral nontargetable CREB expression vector was created as described elsewhere [7]. To rescue CREB expression in stably CREB-silenced cells, A375SM and C8161-c9 CREB-shRNA or NT-shRNA were plated in 6-well plates and transduced with the virus containing either the nontargetable CREB expression vector or empty vector. After 48 h, the cells were replated and selected as described previously [7]. The CREB expression was confirmed by Western Blot.

### Western Blot analysis

CREB, pCREB, E2F-1 and p21*^Waf1^* were detected in total cell extracts (20 µg) while AP-2α was detected in the nuclear fraction (20 µg) by 10% SDS-polyacrylamide gel electrophoresis and transferred to Immobilon P transfer membrane (Millipore). The membranes were washed in Tris-buffered saline with Tween (10 mM Tris-HCl, pH 8, 150 mM NaCl, and 0.05% Tween 20) and blocked overnight at 4°C with 5% nonfat milk in Tris-buffered saline with Tween. The blots were then probed overnight at 4°C with primary antibodies at 1∶2000 (anti-CREB, Cell Signaling), 1∶2000 (anti-pCREB Cell Signaling), 1∶2000 (anti-E2F-1, Santa Cruz), 1∶2000 (anti-p21, Cell Signaling) and 1∶2000 (anti-AP-2α, Santa Cruz). After 2 h of incubation with horseradish peroxide-conjugated secondary antibody, immunoreactive proteins were detected by enhanced chemiluminescence per the manufacturer's instructions (ECL detection system; Amersham Biosciences). For AP-2α in the nuclear extract, A375SM and C8161-c9 were prepared according to the manufacturer's instructions (Nuclear Extraction Kit, Panomics). Protein concentrations were determined by using the Bradford protein assay (Bio-Rad).

### Expression vector constructs

The total RNA was extracted from A375SM and reverse-transcripted (RT) using a commercial kit (Clontech). The pcDNA3.1 for AP-2α was obtained as previously described [10]. The ORF of E2F-1 was PCR-amplified from the RT product with the following two primers: E2F-1-Nhe-F: 5′-CTAGCTAGCATGGCCTTGGCCGGGGCCCCTGC-3′ and E2F-1-R: 5′-TCAGAAATCCAGGGGGGTGAGGTCC-3′. The pcDNA3.1 for nontargetable CREB expression vector was obtained as previously descibed [7]. To create CREB expression vector containing a missense mutation at position 133 (S → A, CREB_(S133A)_), we utilized the following two primers: CreB-S133A-F 5′- CTTTCAAGGAGGCCTGCCTACAGGAAAATTTTG-3′ and CreB-S133A-R 5′-CAAAATTTTCCTGTAGGCAGGCCTCCTTGAAAG-3′. The PCR products were digested with NheI and cloned into pcDNA3.1 through the same restriction enzyme site. The inserted ORF was confirmed by sequencing. Transient transfections were performed using Lipofectamine 2000 (Invitrogen) according to the manufacturer's instructions. As a control, cells were transduced with empty vector pcDNA3.1.

### Reporter constructs and luciferase activity assays

The AP-2α promoter region (nucleotides −1,500 to +50 from the transcription initiation site) was amplified from A375SM cell genomic DNA and ligated into the pGL3-basic vector (Promega). Analysis of transcription factor binding sites was performed using GENOMATIX software (http://www.genomatix.de). Site-directed mutageneses of the CRE sites as well as E2F-1 binding site were performed using the QuikChange II XL Site-Directed Mutagenesis Kit (Stratagene) according to manufacturer's instructions. Transient transfections were performed as previously described [7]. Briefly, a total of 2.5×10^4^ cells/well in a 24-well plate were transfected with 0.8 µg of the basic pGL3 expression vector with no promoter or enhancer sequence or with 0.8 µg of the pGL3-AP-2α firefly luciferase expression constructs. For each transfection, 2.5 ng of cytomegalovirus (CMV)-driven renilla luciferase reporter construct (pRL-CMV, Promega) was included. After 4 hours, the transfection medium was replaced with serum-containing growth medium. After 48 hours, the cells were harvested and subjected to lysis, and the luciferase activity was assayed utilizing a Dual Luciferase Reporter Assay System (Promega) according to the manufacturer's instructions. The luciferase luminescence (relative light intensity ×10^6^) was measured with the LUMIstar reader (BMG Labtech). The ratio of firefly luciferase activity to CMV-driven renilla luciferase activity was used to normalize for differences in transfection efficiency among samples.

### Chromatin Immunoprecipitation assay (ChIP)

ChIP assays were performed utilizing the ChIP-IT Express kit from Active Motif according to the manufacturer's protocol. Briefly, cells were fixed with 1% formaldehyde. The cross-linking reaction was stopped with 0.125 M glycine. The cells were pelleted and resuspended in a hypotonic buffer, and cell nuclei were isolated by using a Dounce homogenizer. The chromatin was then sheared into small fragments by adding an enzymatic solution for 10 minutes at 37°C. Fractions of chromatin solutions were incubated overnight at 4°C with either 3 µg of anti-CREB, anti-E2F-1 or IgG control antibodies crosslinked to magnetic beads. The immune complexes were then eluted from the magnetic beads, and proteins were reverse-crosslinked at 65°C for 2.5 hours. Proteins were digested with 2 µl of Proteinase K at 37°C for 1 hour, extracted in elution buffer, and analyzed by PCR. A 609-bp fragment spanning the −1559 to −850 region of the AP-2α promoter was amplified by PCR using primer sequences forward 5′-GCTCAGCCAGGAAAATTAATTACTCTG-3′ and reverse 5′-CAGGTTGGAGATTCTGCCAAGCGGCTC-3′. Another fragment of 135-bp spanning the −743 to −549 region of the AP-2α promoter was also amplified by PCR using primer sequences forward and 5′-GGTGGTTATGTTTAATTGCGAAAGG-3′ and reverse 5′-GGCATCTCTGGAGAAAAGGAAGC-3′.

### Semiquantitative reverse transcriptase-PCR

One microgram of total RNA was reverse primed with an oligo (dT) primer and extended with Moloney murine leukemia virus reverse transcriptase (Clontech). The PCR was performed using the Clontech Advantage cDNA PCR kit in a 50 µl reaction mixture containing 1x PCR buffer, 5 µl cDNA, 0.2 mmol/L deoxynucleotide triphosphate, and 2.5 units of Taq polymerase. For AP-2α quantification, specific primers (5′-CTGCCAACGTTACCCTGC-3′ and 5′-TAGTTCTGCAGGGCCGTG-3′) were used. Glyceraldehyde-3-phosphate dehydrogenase cDNAs were amplified by PCR in the same reaction mixture and carried out by an initial denaturation for 2 min at 94°C, followed by 27 cycles of denaturation at 94°C for 1 min, annealing at 58°C for 1 min, and extension at 72°C for 1 min, with a final elongation step at 72°C for 5 min. A final elongation step was carried out at 72°C for 10 min.

### Confocal microscopy

Cells (2×10^3^cells) were cultivated on round glass coverslips (13 mm) for 24 h following fixation with 3.7% paraformaldehyde for 15 min at room temperature. Cells were then incubated with block solution (150 mM NaCl (Merck), 50 mM Tris (Gibco Invitrogen, Carlsbad, CA), 0.25% BSA (Sigma), and 0.5% Tween 20 (Sigma), pH 7.2) for 1 hour at room temperature. Anti-AP-2α antibody (5 µg/ml, Santa Cruz) or anti-pCREB antibody (1 µg/ml, Cell Signaling) were incubated for 12 h at 4°C. After several washes in PBS, anti-mouse IgG rhodamine conjugate (1∶500, Sigma) was incubated at room temperature for 1 h. Alternatively, prior to fixation, cells were treated with H-89 (10 µM) for 30 min at 37°C. Staining of nuclei was performed with 50 µg/ml DAPI (Invitrogen) for 1 h at room temperature. The coverslips were treated with a mounting medium (Vectashield; Vector Laboratories, Burlingame, CA) to reduce bleaching and were examined by laser scanning fluorescence confocal microscope (MRC 1024/UV System; Bio-Rad, Hercules, CA) equipped with a transmitted light detector for Nomarski differential interference contrast.

### Soft-Agar colony formation assay

Base layers (2 ml) of Eagle's medium (MEM) supplemented with 20% FBS containing 0.6% seaplaque agarose were set in 6-well plates. This was overlaid with 1 ml of a second layer of 0.8% agar containing a suspension of 5×10^3^ cells/well. All cultures were done in triplicate. Colonies were scored after incubation at 37°C for 25 days.

### Statistical analysis

Significance was determined by a two-tailed Student's *t* test. *p* values <0.05 were considered statistically significant.

## Supporting Information

Figure S1 Inhibition of pCREB results in increased AP-2α expression. Expression of AP-2α was detected in A375SM and C8161-c9 cell lines after incubation with the CaMKIV inhibitor (KN-93), p90RSK inhibitor (SL 0101-1), and AKT inihibitor (Triciribine). Increased AP-2α expression was observed in both cell lines after treatment with each pathway inhibitor. Lamin was used as a loading control.
(0.38 MB TIF)

Figure S2 Inhibition of AP-2α is dependent on pCREB. Transient expression of nontargetable CREB carrying a substitute mutation (S133A) in CREB-silenced cells does not inhibit AP-2α expression in either A375SM or C8161-c9 cell lines. Lamin was used as a loading control.
(0.39 MB TIF)
