# Supplementary figures and images for: CREB Inhibits AP-2α Expression to Regulate the Malignant Phenotype of Melanoma
Source: PLoS One. 2010 Aug 27;5(8):e12452. doi: 10.1371/journal.pone.0012452 (PMC2929203; doi:10.1371/journal.pone.0012452)

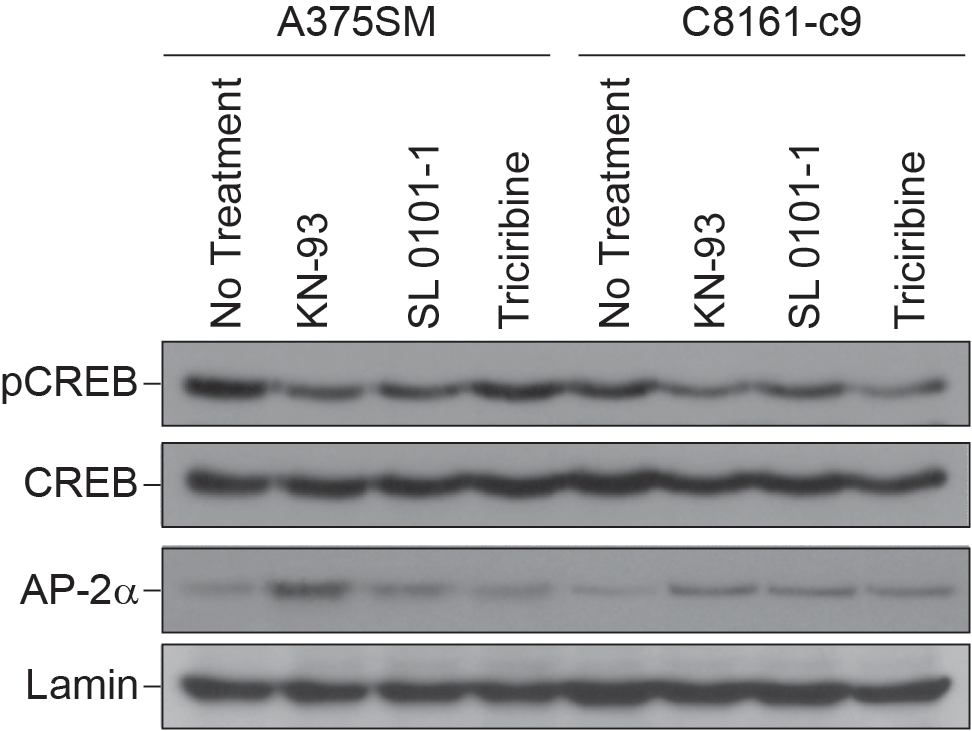

Supplement: Figure S1 — Inhibition of pCREB results in increased AP-2α expression. Expression of AP-2α was detected in A375SM and C8161-c9 cell lines after incubation with the CaMKIV inhibitor (KN-93), p90RSK inhibitor (SL 0101-1), and AKT inihibitor (Triciribine). Increased AP-2α expression was observed in both cell lines after treatment with each pathway inhibitor. Lamin was used as a loading control. (0.38 MB TIF) [file pone.0012452.s001.tif]

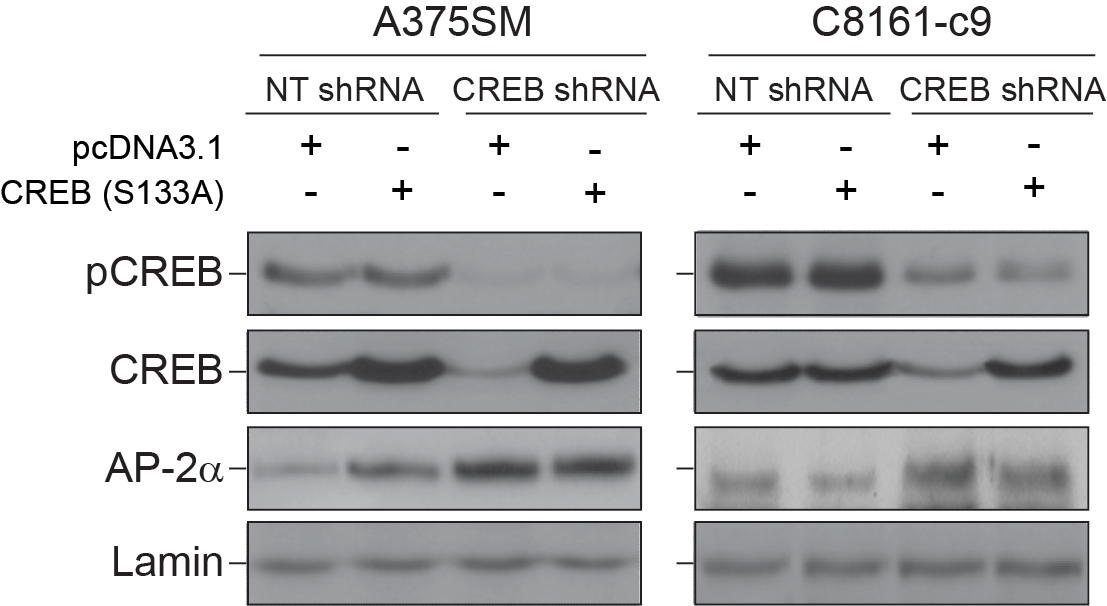

Supplement: Figure S2 — Inhibition of AP-2α is dependent on pCREB. Transient expression of nontargetable CREB carrying a substitute mutation (S133A) in CREB-silenced cells does not inhibit AP-2α expression in either A375SM or C8161-c9 cell lines. Lamin was used as a loading control. (0.39 MB TIF) [file pone.0012452.s002.tif]
